# Adherence to Aromatase Inhibitor Therapy in Breast Cancer: Insights From a Multicenter Italian Study

**DOI:** 10.1155/tbj/8976679

**Published:** 2025-12-19

**Authors:** Maria Agnese Fabbri, Alberto Fulvi, Matteo Vergati, Giuliana D’Auria, Patrizia Vici, Lorena Filomeno, Teresa Arcuri, Antonella Palazzo, Fabrizio Nelli, Cristina Fiore, Ilaria Portarena, Pina Tiziana Falbo, Rosalinda Rossi, Daniele Alesini, Valentina Sini, Roberta Pace, Patrizia Frittelli, Domenico Cristiano Corsi, Lucia Palombi, Simona Pisegna, Andrea Botticelli, Simone Scagnoli, Giorgio Pistillucci, Erica Giordani, Annalisa La Cesa

**Affiliations:** ^1^ UOC Oncologia, Ospedale Belcolle, Viterbo, Italy; ^2^ UOC Oncologia Medica 1, IRCCS Istituto Nazionale Tumori Regina Elena, Roma, Italy; ^3^ UOC Oncologia Interpresidio Pertini/S. Eugenio, Roma, Italy; ^4^ UOSD Sperimentazioni di Fase IV, IRCCS Istituto Nazionale Tumori Regina Elena, Roma, Italy; ^5^ UOC Oncologia medica, Fondazione Policlinico Gemelli IRCCS, Roma, Italy; ^6^ UOC Oncologia Medica, Policlinico Tor Vergata, Roma, Italy, ptvonline.it; ^7^ UOC Oncologia, Azienda Ospedaliera San Giovanni Addolorata, Roma, Italy; ^8^ UOSD Centro Oncologico SSP-NRM, Ospedale Santo Spirito in Sassia, Roma, Italy; ^9^ UOC Oncologia, Ospedale San Camillo de Lellis, Rieti, Italy, provincia.foggia.it; ^10^ UOC Chirurgia Senologica, Ospedale Isola Tiberina Gemelli Isola, Roma, Italy; ^11^ UOC Oncologia Isola Tiberina, Roma, Italy; ^12^ UOC Oncologia San Camillo, Roma, Italy; ^13^ Department of Experimental Medicine, Sapienza University, Roma, Italy, uniroma1.it; ^14^ UOC Oncologia Medica, Ospedale Santa Maria Goretti, Latina, Italy; ^15^ UOC Oncologia Campus Biomedico, Roma, Italy, unicampus.it

**Keywords:** adherence, adjuvant hormonal therapy, aromatase inhibitors, breast cancer, side effects

## Abstract

**Background:**

In estrogen‐receptor positive breast cancer (BC), oral adjuvant endocrine therapy (ET) administered for at least 5 years significantly reduces risks of disease recurrence and mortality. Among available therapies, aromatase inhibitors (AI) showed high efficacy. However, adherence to ET is very poor. Effective support by physicians requires the identification of factors influencing AI treatment adherence.

**Materials and Methods:**

A prospective/retrospective multicentric study was conducted in adult BC women currently undergoing adjuvant treatment with AI. Study endpoints were assessed through a questionnaire after at least 12 months of adjuvant therapy. The primary objective was the assessment of the adherence to AI; secondary objectives were the assessment of adverse events (AEs) of the therapy and the solutions adopted for AEs.

**Results:**

Overall, 903 patients with a median age of 63 years were enrolled. Two hundred and forty‐three patients (26.9%) stated they do not respect the intake times. Adherence was not influenced by the number of drugs other than the ones for BC or by age. Most patients (87%) suffered from one or more AEs. The most frequent are musculoskeletal symptoms, which occurred in 86.2% of the patients. 74.5% and 74.4% of participants reported hot flashes and tiredness, respectively. No structured or uniform responses were reported regarding the strategy for solving side effects: answers were almost generic, but for more than 50% of patients, the final outcome was positive. AEs were a driver for nonadherence in only 19.6% of patients.

**Conclusion:**

Survey results should be considered as an overview of AI therapy adherence in BC patients. We showed that the oncologist has a key role in improving therapeutic adherence and, as a consequence, in improving clinical outcome. Through a dialogue with the patient and a synergistic interaction with other clinical specialists, a greater awareness of the importance of the treatment could be warranted.

## 1. Introduction

Breast cancer (BC) is the most common cancer among women, and BC positive for the estrogen receptor (ER) accounts for over 80% of all BCs [1–4].

Hormone receptor‐expressing (HR+) BC is the most frequent type of BC for which endocrine therapy (ET) is the standard treatment, recommended for 5 years or more, to prevent the development of metastatic disease, loco‐regional recurrences, and contralateral tumors [5].

For many years, tamoxifen, whose action is directed at inhibiting the activation of ER, has been the gold standard for the treatment of HR + BC [6]. More recently, new drugs have been introduced into clinical practice, among them aromatase inhibitors (AI). These new agents inhibit the estrogenic pathway through distinct molecular mechanisms and are able to avoid both side effects such as toxicity on the endometrium and the potential development of resistance to BC treatment itself. Other drugs that act on the estrogenic pathway are ER degraders (e.g., fulvestrant) or inhibitors of the pathways downstream of the receptor (mTOR inhibitors, CDK4/6 inhibitors, PARP inhibitors, and PI3K inhibitors) [7].

Of note, among these new agents, AIs have shown clear benefits and good tolerability [8]. The mechanism of action of AIs, such as exemestane, anastrozole, and letrozole, is the inhibition of the production of the ER ligand, leading to the inhibition of the aromatase enzyme and therefore allowing the reduction of estrogen levels with consequent benefit for BC patients in their treatment journey. While the clinical effectiveness of AIs is widely demonstrated, they are not without adverse events (AEs) either. The most frequent and often severe AEs are joint pain and osteoporosis, which can lead to nonadherence and/or discontinuation of therapy [9].

However, no matter how effective a drug is, its action is compromised when the patient is unable to complete the therapy according to the prescribed dosage in the long term. It is well known how adherence to adjuvant ET decreases from the first to fifth year of treatment. On average, one‐third of patients are not adherent to treatment by the fifth year [8, 10].

This issue is still an unmet medical need. To date, nonadherence (namely, not respecting both dosage and schedule) and nonpersistence (not respecting the recommended duration) correlate negatively with overall survival, event‐free survival, and distant disease‐free survival [11, 12]: it is therefore necessary to set in to identify the reasons for treatment nonadherence and the strategies which must be adopted in order to improve patients’ outcomes.

The factors underlying nonadherence and nonpersistence to ET are difficult to identify, and their evaluation depends on the method used and the time frame analyzed [13]. Yussof and colleagues identify 19 recurring factors significantly associated with long‐term adherence, which, according to Toivonen and colleagues, can be grouped into six categories: (1) social and economic factors; (2) factors related to treatment and to the burden of disease; (3) psychological factors; (4) factors related to the healthcare system; (5) factors related to treatment and AEs; and (6) factors related to quality of life (QoL) [8, 13]. There is therefore an essential need to identify follow‐up routines to support BC women on ET and to enable compliance to ET, preventing drug discontinuation. How follow‐up should be conducted to optimize compliance is at present not known. Further research using objective or multiple measures of treatment adherence is needed to improve the validity of results.

Unfortunately, adherence and persistence to ET is often suboptimal among BC patients, also in younger patients [14]. Women commonly felt isolated and neglected as a result of insufficient information and support from healthcare personnel. If women are to persist with ET, primary care providers should be aware of the facilitators and barriers to adherence, and they should be knowledgeable in symptom management strategies [15].

This national multicentric project aimed to outline the causes of nonadherence in a real‐life setting and the possible paths to be taken to improve both QoL and the clinical outcome of the patient BC patients undergoing ET with effective interventions by the treating oncologists.

## 2. Materials and Methods

### 2.1. Study Design and Participants

A prospective/retrospective multicentric study was conducted between May and September 2023 in 13 Italian Oncologic Centers on women aged 18 years or older with histologically confirmed diagnosis of advanced BC (ER and PgR positive [> 10%] and HER2 negative) undergoing adjuvant treatment with AI (and they should have been undergoing treatment for at least 12 months). Previous or concomitant treatment with bisphosphonates and denosumab was allowed.

### 2.2. Study Objectives

The primary objective was the assessment of the adherence to AI therapy.

Secondary objectives were the assessment of AEs of the therapy and the description of the solutions adopted for AEs solutions and, if applicable, their outcomes.

The final aim was to identify the various issues in the therapeutic pathway to also provide possible effective strategies in improving treatment adherence and persistence.

Study endpoints were assessed through an anonymized validate self‐filled questionnaire, completed after at least 12 months of adjuvant ET therapy. Adherence was measured taking into account physician prescription and patients’ answers to the questionnaire. In detail, regarding treatment adherence, we asked patients if they have difficulty remembering to take drugs, how many other drugs they take, if the packaging of the active agent is always the same, and, if the packaging changes, which professional figure has decided on this change and, in case of emerging doubts on the therapy, who is patients’ primary professional healthcare reference personnel. Regarding side effects, we asked for the type and frequency of musculoskeletal symptoms, hot flashes and tiredness, weight gain, hair loss, and sexual disorders. We also recorded if side effects/symptoms influenced the correct drug assumption and, in case of holding, if the patient has advised the treating physician. If the patient has changed packaging, we ask if a difference in side effects occurred.

Finally, the questionnaire was focused on the type and quality of the relationship with the treating physician, and, particularly for side effects regarding sexual disorders on how they were solved (if applicable) or tackled.

This study complied with the standards of the Helsinki Declaration. All patients were enrolled after providing written informed consent. The study was approved by our institutional board (Ethical Committee AVEC of Lazio 1, approval ID 333/CE).

### 2.3. Statistical Analysis

Starting from a survey shared with the Steering Committee of the project, the questionnaire was developed. Inside the promoting center of the study, there is a consulting office responsible for the validation of the questionnaires (test/re‐test technique with Pearson correlation coefficient). Demographics and patients’ characteristics were summarized by descriptive statistics. No power analysis was performed since the questionnaire was proposed to all the patients who met the inclusion criteria in the enrollment period. Patients were consecutively enrolled to avoid selection bias. Regarding inferential statistics, comparisons between groups were performed—for categorical variables—using the contingency table analysis with the chi‐squared or Fisher’s exact test, as appropriate, whereas continuous data were analyzed using a Student′s *t*‐test, after checking whether data are normally distributed (based on the Shapiro–Wilk statistic), or a Wilcoxon rank‐sum test otherwise. All tests were two‐sided, and a *p* value of less than 0.05 was considered statistically significant. Statistical analyses were performed with Stata 17 (StataCorp LP, TX).

## 3. Results

### 3.1. Patients’ Characteristics

Overall, 903 questionnaires were collected from 903 patients with a median age of 63 years (range 28–91, interquartile range 55–72) and an average duration of treatment with adjuvant ET of 2.5 years (range 12–60 months). All patients were on AI treatment at the time of interview. 68% (614 out of 903) of the women interviewed were found to be treated with generic drugs, 24% (*n* = 217 out of 903) with branded drugs, and 8% with originator drugs. In addition to ET for BC, most patients (96%) take drugs for other medical conditions. Among them, 40% of patients (*n* = 347) take more than two tablets, a trend increasing with patients’ age.

Thirty‐seven percent of the women interviewed were unable to state the name of the drug or how long they have been taking ET.

### 3.2. Adherence

**Figure 1:**
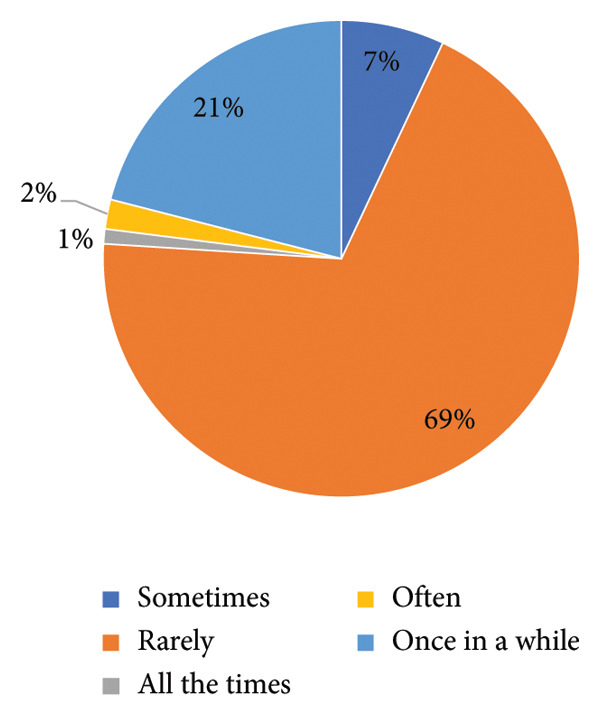
Answers to the question “How many times do you forget to take drugs?” in patients changing packaging.

Two hundred and forty‐three out of 903 patients (26.9%) stated they do not respect the intake times. Data analysis on the impact of packaging changes on treatment adherence reveals that 28.9% (271 out of 903) of patients receive different packaging for the active agent. Among these patients, 24% report that the substitution is determined by the oncologist, 48.1% attribute it to pharmacy availability, and 18.1% indicate that the decision is made by the general practitioner (Table 1). More than 60% of patients are unable to report the reason for changing medication, but 96% of the women interviewed declared that they have never stopped taking the drug without informing their physician. In detail, 69% of women who change packaging respect the intake times, whereas the other 31% reported that they forget to take the drug once in a while (*n* = 56, 20.7%), sometimes (*n* = 18, 6.6%), often (*n* = 6, 2.2%), or all the time (*n* = 4, 1.5%) (Figure 1). On the other hand, adherence was not influenced by the number of drugs other than the ones for BC used by the patients (*p* = 0.778) or by age (≥ 70 years vs. < 70 years, *p*  > 0.999).

**Table 1:** Questionnaire, adherence items.

Question	*n* (%)^∗^
Taking medications every day can be uncomfortable. Have you difficulty remembering to take your drugs?	660 (73.1)
Rarely	140 (15.5)
Once in a while sometimes	63 (67.7)
Often	14 (1.6)
All the times	26 (2.9)
How many medications do you take in addition to the BC drug?	
0‐2	535 (59.2)
3‐5	261 (28.9)
> 5	107 (11.8)
Do you always use the same package or even different packages of the same active agent?	
Yes	632 (70.0)
No	271 (30.0)
Is the replacement due to? (*n* = 287)	
Oncologist	83 (28.9)
General practitioner	52 (18.1)
Pharmacist	138 (48.1)
All the three	14 (4.9)
If you have doubts about cancer therapy, who do you ask for advice?	
Oncologist	741 (82.1)
General practitioner	146 (16.2)
Pharmacist	9 (0.9)
All the three	7 (0.8)

^∗^where not otherwise specified the % were calculated on 903 responses.

### 3.3. AEs

The responses collected indicated that side effects of AI treatment were a driver for nonadherence in only 19.6% of patients (*p* = 0.668). Regarding this aspect, the responses obtained showed that most patients (87%) suffered from one or more AEs. Among these, the most frequent are musculoskeletal symptoms, which occurred in 86.2% of the patients interviewed. 74.5% and 74.4% of participants reported hot flashes and tiredness, respectively. Tiredness was the most common cause of interruption in older patients (> 70 years).

61.8% of patients experienced weight gain. Other side effects that have emerged are hair loss, reported in 57.4% of patients and, slightly less frequently, sexual disorders (51.6%) (Table 2).

**Table 2:** Questionnaire, adverse events items.

Question	*n* (%)
Hot flashes, weight gain, musculoskeletal symptoms, hair loss, tiredness and sexual disorders are common adverse events during treatment with endocrine therapy. Have you ever experienced one or more of the symptoms mentioned above? (*n* = 861)	
Yes	746 (86.6)
No	115 (13.4)
Hot flashes^∗^	153 (20.5)
Rarely	98 (13.1)
Once in a while sometimes	121 (16.2)
Often	115 (15.4)
All the times	69 (9.2)
Weight gain^∗^	140 (18.8)
Rarely	79 (10.6)
Once in a while sometimes	87 (11.7)
Often	78 (10.5)
All the times	77 (10.3)
Musculoskeletal symptoms^∗^	79 (10.6)
Rarely	67 (9.0)
Once in a while sometimes	174 (23.3)
Often	169 (22.7)
All the times	154 (20.6)
Hair loss^∗^	223 (29.9)
Rarely	59 (7.9)
Once in a while sometimes	77 (10.3)
Often	47 (6.3)
All the times	22 (2.9)
Tiredness^∗^	97 (13.0)
Rarely	92 (12.3)
Once in a while sometimes	150 (20.1)
Often	123 (16.5)
All the times	93 (12.5)
Sexual disorders^∗^	132 (17.7)
Rarely	37 (5.0)
Once in a while sometimes	81 (10.9)
Often	86 (11.5)
All the times	59 (7.9)

^∗^The percentages were calculated according to the number of patients who declared at least one symptoms (*n* =746).

Regarding AEs perceived in the sexual sphere, the first data that emerges from the survey is that there is discomfort, especially among older women, in talking about this topic, with a third of patients not answering the question on the frequency of disorders, 25% saying they are unable to discuss it with the oncologist, and 84% not providing an answer regarding how these issues were addressed.

As regards the influence of the switch between generic drugs can have on AEs reported in the previous paragraph, the questionnaire shows that only 9% of patients perceived differences in side effects due to the change in packaging.

Most patients (90%) reported that they refer to the oncologist for any doubts about the therapy, and 97% stated that they have good communication with the clinical specialist.

No structured or uniform responses were reported in the open question regarding the strategy for solving or attenuating side effects: answers were almost generic since it was a free text answer field, but for more than 50% of patients, the final outcome was a positive one.

## 4. Discussion

Nonadherence to ET is a complex problem whose reasons can be found in various areas ranging from inconveniences linked to the side effects to lack of awareness of the importance of the therapy. It is essential, therefore, to identify patients at risk of nonadherence and to identify the factors that may hinder adherence to treatment. In line with the data present in the literature, 27% of our study patients stated they do not respect the intake times [8, 13].

AEs represent an inevitable component of treatment and a factor frequently associated with nonadherence. The correct management of AEs therefore represents a fundamental element on which to intervene, both through the adoption of preventive measures and through a multidisciplinary approach that involves various professional figures, including gynecologists, psychologists, physicians specializing in arthralgia and osteoporosis, and specialists in physical activities for cancer patients.

Sequential AI‐based treatment and treatment with AI alone are generally associated with a greater probability of adherence, while frequent substitutions between generic drugs are correlated with lower adherence and persistence and can influence the efficacy and safety of the treatment. The change of packaging between generic drugs therefore represents an important problem, since it can determine a significant variability in exposure to the drug, but it is a problem difficult to manage, as it would require an intervention on the territory. Precise indications on prescriptions to stick to the therapy and the prescribed packaging can be a solution to this problem. More than 60% of the patients are unable to report the reasons for changing medication, which indicates that patients have poor awareness of the therapy they are taking. Although our study did not find an association between adherence and polypharmacy, regardless of the number of dispensed drugs, the available evidence on this issue is contradictory [16]. In a systematic review assessing adherence to treatment with oral antineoplastic drugs, seven studies showed that polypharmacy was associated with lower adherence, and four concluded in the opposite way [17]. An explanation for the discrepancy among the studies may be the lack of a consensus on the definition of polypharmacy [18]; however, our conclusions on adherence to therapy were not influenced by the number of drugs used by patients.

Adherence to antihormonal therapy for BC is confirmed to be suboptimal, especially among patients with a relatively good prognosis. Nonadherent patients tend to discontinue their antihormonal therapy in the initial part of the treatment period. Several studies report, in fact, that about 17%–25% of patients do not follow the prescriptions from the beginning while, during treatment, adherence to AI treatment varies from 72% to 80%, respectively [19]. Thus, targeted interventions to improve adherence should be mostly focused on the first part of the treatment period [20]. Of note, 96% of the women interviewed declared that they have never stopped taking the drug without informing their physician, labeling a good dialogue with their oncologist who can promptly intervene, also involving other clinicians. In fact, in recent years, synergistic use of different disciplines led to improvement in endocrine‐related and overall QoL among BC survivors who were experiencing side effects from AIs, for example, other combined aerobic and resistance exercises, such as treadmill walking and strength training. Because AEs associated with AI assumptions are common and are the main cause for treatment discontinuation, nonpharmacologic intervention could benefit patients and increase successful adherence to AIs in BC settings [21]. Nevertheless, in our study, AEs were a driver for nonadherence in only 19.6% of patients. Unfortunately, since the question on how the side effects were addressed was open‐ended, we were unable to obtain a clear picture of the situation. This will serve as a point for improvement for future research and daily clinical practice.

### 4.1. Conclusion

In this study, we wanted to explore the natural history of adherence to ET in a real‐life setting. Intervening on the patient to improve adherence to therapy emerges as the best strategy to pursue and where the margin for intervention is widest. Since the oncologist stands out from the survey as a reference figure for patients, it appears that the physician has a key role in improving therapeutic adherence, and therefore clinical outcome, with a concrete possibility of being able to create, through dialogue with the patient and a synergistic interaction with other clinical specialists, a greater awareness of the importance of the treatment.

## Consent

This study involved human participants. The patients received oral and written information, and all gave their written informed consent to the study.

## Conflicts of Interest

S.S. received fee as speaker from Lilly, Novartis, Pfizer, MSD, BMS, Pierre Fabre, Daichi Sankyo, Astrazeneca; P.V. received fee from Lilly, Pfizer, Novartis, Daiichi‐Sankyo, MSD, Gentili; L.F. received fee from Daiichi‐Sankyo, Eisai, Gentili. The other authors declare no conflicts of interest.

## Funding

No funding was received for this manuscript.

## Data Availability

Data that support the findings of this study are available on request from the corresponding author. The data are not publicly available due to restrictions, for example, they contain information that could compromise the privacy of the study participants.
